# The effect of lemon inhalation aromatherapy on pain, nausea, as well as vomiting and neurovascular assessment in patients for lower extremity fracture surgery: a randomized trial

**DOI:** 10.1186/s12906-023-04047-z

**Published:** 2023-06-24

**Authors:** Masoume Rambod, Nilofar Pasyar, Zahra Karimian, Arash Farbood

**Affiliations:** 1 Community Based Psychiatric Care Research Center, Nursing and Midwifery School, Shiraz University of Medical Sciences, Shiraz, Iran; 2 Student Research Committee of Shiraz University of Medical Sciences, Shiraz, Iran; 3 Anesthesiologist and Pain Specialist, Shiraz Anesthesiology and Critical Care Research Center, Shiraz, Iran

**Keywords:** Aromatherapy, Lemon oil, Pain, Postoperative Nausea and Vomiting

## Abstract

**Background:**

Complementary and integrative medicine may be effective for postoperative outcomes. This study aimed to determine the effect of lemon inhalation aromatherapy on pain, nausea, and vomiting and neurovascular assessment in patients for lower extremity fracture surgery.

**Methods:**

This is a randomized clinical trial study. Ninety patients who had undergone lower extremity fracture surgery were randomly assigned to the intervention (lemon aromatherapy) and control groups. Lemon aromatherapy was started in the morning of the surgery and extended at two-hour intervals until the end of the surgery, in the recovery room, and 16 h after surgery. Numerical pain and nausea and vomiting scales, the Rhodes Index of Nausea, Vomiting, and Retching, and the WACHS Neurovascular Observation Chart were used to assess the outcomes before and after the intervention (in the recovery room and 4, 8, 12, and 16 h post-surgery). The data were analyzed using the Wilcoxon test, ANCOVA, and Repeated Measure ANCOVA.

**Results:**

A significant difference was observed between the groups in terms of the intensity of pain (*P* < 0.001) and nausea and vomiting (*P* = 0.001) during the study period. Moreover, a significant difference was found between groups as to the frequency and severity of nausea, vomiting, and retching. The amount and duration of postoperative vomiting and nausea were significantly lower in the intervention group compared to the control group. In addition, lemon inhalation aromatherapy decreased the frequency of anti-emetic drug administration in the recovery room (*P* = 0.04) and 16 h post-surgery (*P* = 0.03).

**Conclusions:**

This study indicated that aromatherapy reduced pain intensity, postoperative nausea, vomiting, and retching, as well as the incidence of anti-emetic drug administration. Therefore, using lemon inhalation aromatherapy to relieve pain and reduce nausea and vomiting is suggested for lower extremity fracture patients who have undergone surgery.

**Trial registration:**

This study was registered in the Iranian Registry of Clinical Trail (Number = 57,331, IRCT20130616013690N10, approved 24/07/2021) (https://www.irct.ir/trial/57331).

## Background

Lower extremity fractures are associated with severe disability, recovery, and long-term treatment and require the most surgical procedures [1]. It was reported that mild to severe pain is common after orthopedic surgery [2]. A study reported that 98.4% of patients who had undergone orthopedic surgery experienced pain [3]. Postoperative persistent pain might have negative consequences for physical and mental health. For example, it can lead to hypoventilation, an increase in oxygen demand, and a loss of daily living activities that might even turn into chronic pain, sleep disturbances, patients' anxiety, and dissatisfaction with life [4]. Pain management after orthopedic surgery is more challenging than other types of surgery [2]. Drugs are commonly used to relieve postoperative pain in lower-limb fractures. Although narcotics are very effective in controlling postoperative pain, they also cause side effects such as drowsiness, ileus, constipation, inhibition in the respiratory and central nervous systems, addiction, and nausea and vomiting [5].

Postoperative nausea and vomiting are among the common post-surgery side effects. Literature showed that one third of postoperative patients experienced moderate to severe nausea and vomiting following general anesthesia [6]. Nausea and vomiting are also the second most commonly reported complaint following spinal anesthesia [7]. In a study on orthopedic patients who had undergone surgery, 59.3% and 39.0% of patients reported nausea and vomiting, respectively [3]. In another study, it was indicated that 0 to 6 h after hip and knee surgeries, 73% and 27% of patients had nausea and vomiting, respectively [8]. These showed that postoperative nausea and vomiting is common after orthopedic surgery [9].

Swelling and pressure on the muscles and arteries after a fracture are dangerous and may cause complications such as compartment syndrome [10]. Neurovascular assessment is essential for early detection of vascular and nerve damage because delay in diagnosis may lead to permanent defects, limb loss, and even death [11].

As previously stated, pain, nausea, and vomiting are common, and so are neurovascular complications; thus, providing some complementary and integrative health (CIH) may be essential for these patients. The effectiveness of aromatherapy in the treatment of postoperative nausea and vomiting was reviewed in many studies [6, 12]. In a study, it was reported that CIH was effective in reducing the incidence of nausea and vomiting [12]. It was indicated that aromatherapy with lavender and citrus aurantium relieved pain in conscious patients in an intensive care unit [13]. The antinociceptive effect of lemon aromatherapy was shown in mouse postoperative pain [14]. In another study, the antioxidant and antinociceptive effects of lemon citrus in mice were reported [15]. In pregnant women, lemon aromatherapy reduced nausea and vomiting [15]. In fact, lemon had anti-inflammatory, antimicrobial, and anticancer activities [16].

As mentioned, complications such as pain, nausea and vomiting, and neurovascular impairment are common in patients with lower extremity fractures. Therefore, CIH may relieve postoperative pain and subsequently improve physiological parameters, rehabilitation, and initial mobility, reducing the need for analgesics. Previous studies on the effect of inhaled lemon aromatherapy in patients with fractures are limited [14]; therefore, to improve the evidence-based practice, the present study aimed to determine the effect of lemon inhalation aromatherapy on pain, nausea, and vomiting as well as neurovascular assessment in patients for lower extremity fracture surgery.

## Methods

### Design

This is a clinical trial study with a parallel group. The study was registered in the Iranian Registry of Clinical Trail (Number = 57,331, IRCT20130616013690N10, approved 24/07/2021) (https://www.irct.ir/trial/57331).

### Settings

This study was done in the orthopedic clinical treatment rooms, operating rooms, and recovery rooms of the Shiraz University of Medical Sciences hospitals Namazi and Rajaie.

### Eligibility criteria for participants

The participants of this study were the patients admitted to the hospital for lower extremity fracture surgery. These fractures consisted of the hip, femur, tibia, fibula, ankle, and heel. The inclusion criteria were being 18 years old or older, having lower limb fracture surgery, being admitted to the hospital before the orthopedic surgery, and being oriented to time, person, and place. The patients who had asthma and respiratory allergies or were sensitive to extracts of the plant, had olfactory problems and nasal injuries, were known cases of psychological disorders (major depression, anxiety, psychosis, etc.), participated in previous studies, received complementary and alternative interventions such as aromatherapy, meditation, relaxation, massage, acupuncture, etc. a week before the intervention, and had COVID-19 were excluded. Moreover, the patients who had compartment syndrome before the intervention were excluded.

**Fig. 1:**
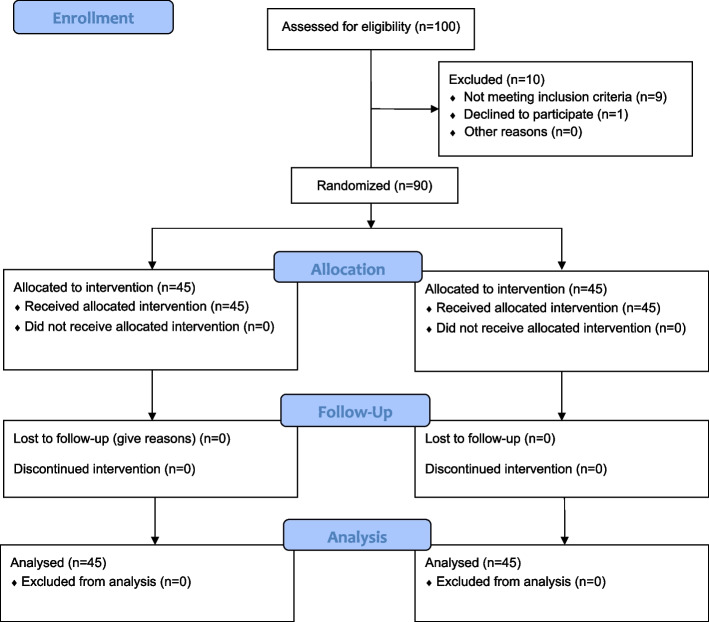
Flow Diagram of participants in this study

At first, one hundred patients participated in this study. Nine patients did not meet the inclusion criteria, and one of them was unwilling to participate, so 90 patients participated and were randomly assigned to the intervention and control groups. All of them continued the study and finished it (Fig. 1).

### Sample size

The sample size in this study was based on a pilot study. Based on pain severity μ1-μ2 = 1.7, SD = 0.9 and drop rate = 20%, the sample size was estimated 90 patients (45 in each group). Moreover, based on nausea and vomiting severity μ1-μ2 = 4, SD = 3 and drop rate = 15%, the sample size was determined 52 patients (26 in each group). Therefore, regarding the higher sample size required, it was determined as 90 patients (45 in each group).

### Randomization

As to randomization, the following were performed: Firstly, Rajai and Namazi hospitals were selected as two separate strata. Then, using WinPEPI software and balanced randomization of separate strata, a list of random allocations was set for 90 patients. Therefore, according to the list in each stratum (hospital), 45 patients were randomly divided into the intervention or control groups. A statistician who was not one of the research team generated the random allocation sequence using WinPEPI software and made sequentially numbered containers. A researcher assistant assigned the participants to the intervention based on random sequence that was in sequentially numbered containers.

### Blinding

In order to conduct blinding, the assistant researcher who collected the data as well as the statistician who analyzed them were blinded to the groups and the assignment of individuals in the groups. Moreover, clinicians and nurses in the orthopedic clinical treatment rooms, and recovery and operation rooms were blind to the groups.

### Outcomes

The outcomes of this study were pain, nausea, and vomiting and neurovascular index. The variables were measured before the intervention, on entering the recovery room, 4, 8, 12, and 16 h after entering the orthopedic surgery ward. Pain intensity was assessed by a numerical rating scale. It numbered 0 to 10, which represented “no pain” to “the worst pain ever possible”, respectively. In this study, the test–retest score of the scale was found to be 0.94.

Post-operative nausea and vomiting were measured using a numerical rating scale and the Rhodes Index of Nausea, Vomiting, and Retching. A numerical rating scale was used to rate the itemsfrom zero to 10. Higher scores indicated a higher intensity of nausea and vomiting. The Rhodes Index of Nausea, Vomiting, and Retching also measured the severity (no, mild, moderate, great, and severe) and frequency (no, 1–2, 3–4, 5–6, and seven or more) of these variables. This index measured the amount of vomiting (I did not throw up, small (up to ½ cup, moderate (½-2 cups), large (2–3 cups), and very large (3 cups and more)) and the duration of nausea (not at all, 1 h or less, 2–3 h, 4–6 h and more than 6 h) [17]. This index assessed the status of the patients’ nausea, vomiting, and retching in the last 16 h. The reliability of the Persian version of this index is approved by Cronbach’s alpha = 0.88 [18]. In this study, the reliability of the index using Cronbach’s alpha was approved (0.96).

The WACHS Neurovascular Observation Chart was used to assess vascular, neurological, and compartment syndrome signs in the lower extremity under surgery. In vascular assessment, skin color and temperature, capillary refill times, and distal pulse were observed. For neurological assessment, movement, and sensation of the lower extremity under surgery were observed. In compartment syndrome assessment, the lower extremities under surgery were assessed for “pain on passive movement, increasing pain not relieved by analgesia, pain at rest, and edema”.

### The inhalation aromatherapy protocol

In the intervention group, 5 drops of lemon essence was put on the patient's surgical facemask when the patients were in orthopedic clinical treatment rooms until he/she was transferred to the operation room. In the operation room and during orthopedic surgery, it was poured on a cotton ball and attached to the participants’ clothes with a distance 20 cm from the patient's nose. In the recovery room or post-anesthesia care unit (PSCU), it was poured on a cotton and inserted in the upper and right edge of oxygen mask which covered the patients’ nose and mouth. In the control group, bitter almond oil, which was odorless, was used in a similar way to the intervention group. A researcher assistant conducted the intervention. In fact, aromatherapy was done continuously. That is, the essential oil was poured on the surgical mask or cotton in each of the groups and inserted in the desired location. Cotton or surgical mask was replaced every two hours with essential oil aromatherapy. Aromatherapy was started in the morning of the lower limb fracture surgery and extended at two-hour intervals until [19] the end of the surgery, in the recovery room or PSCU, and 16 h after surgery. As known, pain, nausea, and vomiting are common within 24 h following a surgical procedure. The length of orthopedic operation surgery and recovery room are 1˗4 and 1˗2 h, respectively. Therefore, aromatherapy with lemon essential oil was started before the surgery and continued until 16 h after the surgery. This time was approximately 24 h for all patients.

The lemon essence was a product of Barij Essence Pharmaceutical Co. and the certificate of decomposition of it was approved and delivered by the quality control unit. In fact, the production and analysis process of lemon essential oil was conducted by Barij Essence Pharmaceutical Co. To analyse lemon essential oil, we used a gas chromatography–mass spectrometry device (Agilent technologies model 6890). Analysis of lemon essential oil showed that the major components consisted of limonene, β- terpinene, γ terpinene, β-caryophyllene, neral, α-terpineol, neryl acetate, geranial, and geranyl acetate.

Limited information has been reported on the side effects of lemon essence. It was reported that distilled sour lemon essence was not phototoxic. Unsolved essence irritated rabbits and mice moderately. However, a previous study showed that lemon essence had a lower risk of phototoxicity and that it was not allergenic [20]. In a study by Rambod et al., it was indicated that this essence made by the mentioned company was not allergic, and no side effects were reported [19]. The possible side effects were explained to the patients, and they were asked to contact the researcher in case of any side effects. The study should be discontinued if any aromatherapy side effects occur.

### Ethical consideration during preoperative, intraoperative and postoperative stages

This study has been approved by the Research Ethics Committees of the School of Nursing and Midwifery, Management and Medical Information Science at Shiraz University of Medical Sciences (IR.SUMS.NUMIMG.REC.1400.001, approval date: 2021–06-08). The informed consent form was signed by all the participants. The patients were informed about the objectives, duration of the study, and side effects. They also were ensured that participating or not participating in the study was optional and that they could withdraw from the intervention whenever they wanted.

The data were analyzed using SPSS software version 26. In this study, the type of anesthesia (general or spinal), site of fracture, use of analgesics in pre-, intra-, and post-operative stages, use or non-use of anti-emetic drugs in vomiting in recovery and in the post-operative ward were considered as confounding variables. The data were analyzed using the Wilcoxon signed-rank test, ANCOVA, Chi-square, and Repeated Measure ANCOVA.

## Results

Ninety patients participated and were randomly assigned to the intervention and control groups. All of them continued the study and finished it. The patients participated in this study before the intervention, on entering the recovery room, 4, 8, 12, and 16 h after entering the orthopedic surgery ward. No side effect, harm, or unintended effects were reported because of the intervention.

The mean age of the patients was 38.06 (SD = 13.98), and 43.77 (SD = 14.16) in the intervention and control groups, respectively. Moreover, 38 (84.4%) patients in the intervention and 37 (82.2%) in the control groups were male. Moreover, 23 (51.1%) and 25 (55.6%) participants in the intervention and control groups underwent spinal anesthesia, respectively, and the others underwent general anesthesia. In the intervention group, 3 (6.7%), 24 (53.3%), 15 (33.3%), and 3 (6.7%) had ankle, tibia, femur, and hip fracture, respectively. In the control group, 5 (11.1%), 22 (48.9%), 15 (33.3), and 3 (6.7%) had ankle, tibia, femur, and hip fracture, respectively. This study showed both groups were homogenous regarding the age, gender, types of anesthesia during surgery, and site of fracture.

**Fig. 2:**
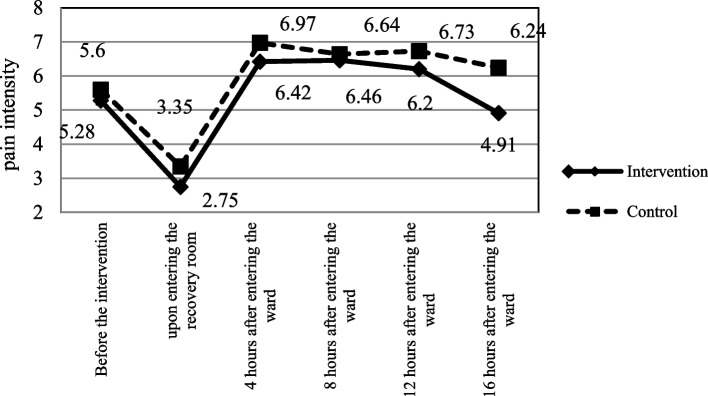
Pain intensity in the intervention and control groups during the study period

Before the intervention, no significant difference was reported between the intervention and control groups in terms of pain intensity (F = 1.88, *p* = 0.17). Also, no significant difference was found between the two groups with regard to pain intensity 8 h after entering the orthopedic ward (F = 1.16, *p* = 0.28). On the other hand, a significant difference was indicated between the two groups with respect to pain intensity upon entering the recovery room and 4, 12, and 16 h post-surgery. Repeated measure ANCOVA showed a significant difference between the groups in terms of pain intensity during the study period (F = 23.89, *p* < 0.001) (Table 1 and Fig. 2).

**Table 1:** Comparison of pain, nausea and vomiting and number and dose of painkiller administration in the intervention and control groups during the study period

Variables	Times	Groups		Test, *p*-value
		Intervention Mean (SD)	Control Mean (SD)	
Pain intensity	Before the intervention	5.28 (0.94)	5.60 (1.26)	F = 1.88, *p* = 0.17^a^
	Entering recovery room	2.75 (2.99)	3.35 (3.17)	F = 4.45, *p* = 0.03^b^
	4 h after entering the ward	6.42 (1.11)	6.97 (1.19)	F = 5.43, *p* = 0.02^c^
	8 h after entering the ward	6.46 (0.91)	6.64 (0.98)	F = 1.16, *p* = 0.28^c^
	12 h after entering the ward	6.20 (0.91)	6.73 (1.13)	F = 6.49, *P* = 0.01^c^
	16 h after entering the ward	4.91 (0.82)	6.24 (0.88)	F = 65.88, *p* < 0.001^c^
	Between groups ANCOVA repeated measure	F = 23.89, *p* < 0.001^c^		
Number of painkiller administration	24 h before the intervention	1.82 (0.64)	1.73 (0.57)	t =—0.68, *p* = 0.49
	In the recovery room	0.46 (0.69)	0.44 (0.62)	t =—0.16, *p* = 0.87
	16 h after surgery and in the ward	2.80 (0.54)	2.60 (0.53)	t =—1.74, *p* = 0.08
	Between groups repeated measure	F = 1.60, *p* = 0.20		
Dose of pethidine administration	16 h after surgery and in the ward	27.29 (7.22)	30.15 (10.11)	t =—1.36, *p* = 0.17
Nausea and vomiting intensity	Before the intervention	–––––	–––––	––––
	Entering recovery room	0 (0.0)	5.75 (0.95)	F = 5.06, *p* = 0.02^d^
	4 h after entering the ward	0.11 (0.55)	1.45 (2.31)	F = 7.92, 0.006^e^
	8 h after entering the ward	0 (0.0)	0.54 (1.82)	F = 0.72, 0.39^e^
	12 h after entering the ward	0 (0.0)	0.06 (0.45)	F = 0.07, 0.78^e^
	16 h after entering the ward	0 (0.0)	0.04 (0.30)	F = 0.07, 0.78^e^
	Between groups ANCOVA repeated measure	F = 12.94, *p* = 0.001		

^a^The use of analgesics during last 24 h, and the site of fracture as covariates

^b^The type of anesthesia, the use of analgesics in pre-intra operative, and the site of fracture as covariates

^c^The type of anesthesia, the use of analgesics in pre-intra and postoperative, and the site of fracture as covariates

^d^The type of anesthesia and site of fracture as covariates

^e^The type of anesthesia, site of fracture, and the use or non-use of anti-emetic drugs in vomiting in recovery and in the postoperative ward as covariates

No significant difference was observed between the groups in terms of the number of painkillers during the 24 h before the intervention (t =—0.68, *p* = 0.49). This variable was not different between the intervention and control groups in the recovery room and 16 h after entering the orthopedic ward (*p* < 0.05) (Table 1). During the 16 h postoperative in the ward, 34 (75.6%) and 35 (77.8%) patients used opioid drugs such as pethidine in the intervention and control groups, respectively, and the other patients used non-opioid medicine such as paracetamol in both groups. Both groups were similar regarding the types of opioid vs. non-opioid medication (χ^2^ = 0.06, *p* = 0.80) and dose of pethidine administration (t =—1.36, *p* = 0.17) (Table 1) during the 16 h postoperative in the orthopedic ward.

A significant difference was observed between the intervention and control groups with regard to numeric nausea and vomiting scale upon entering the recovery room, on entering the orthopedic ward, and 4, 8, 12, and 16 h after entering the ward (*p* < 0.05) (Table 1). A clinically valuable finding was that the intensity of nausea and vomiting upon entering the recovery room, 8, 12, and 16 h after entering the ward were all zero.

A significant difference was observed between the groups in terms of frequency and severity of nausea, vomiting and retching, amount of vomiting, and duration of nausea during the study period (Table 2). Moreover, this study showed the frequency of administration of anti-emetic drugs was significantly lower in the lemon essence aromatherapy group compared to the control group in the recovery room and orthopedic ward (*p* < 0.05) (Table 2).

**Table 2:** Comparison of Rhodes index of nausea, vomiting and retching and anti-emetic drugs administration in the intervention and control groups during the study period

**Variables**	Groups		χ^2^, *p*—value
	Intervention n (%)	Control n (%)	
**Severity of nausea**			
No	44 (97.8)	30 (66.7)	15.14, 0.002
Mild	1 (2.2)	7 (15.6)	
Moderate	0 (0.0)	7 (15.6)	
Great & Severe	0 (0.0)	1 (2.2)	
**Severity of vomiting**			
No	43 (95.6)	33 (73.3)	10.51, 0.01
Mild	2 (4.4)	3 (6.7)	
Moderate	0 (0.0)	8 (17.8)	
Great & Severe	0 (0.0)	1 (2.2)	
**Severity of retching**			
No	45 (57.0)	34 (75.6)	12.53, 0.006
Mild	0 (0.0)	3 (6.7)	
Moderate	0 (0.0)	7 (15.6)	
Great & Severe	0 (0.0)	1 (2.2)	
**Frequency of nausea **^**a**^			
No	42 (93.3)	30 (66.7)	13.14, 0.004
1–2	3 (6.7)	4 (6.7)	
3–4	0 (0.0)	7 (15.6)	
5 or more	0 (0.0)	4 (8.9)	
**Frequency of vomiting **^**a**^			
No	45 (57.0)	34 (75.6)	12.53, 0.006
1–2	0 (0.0)	6 (13.3)	
3–4	0 (0.0)	4 (8.9)	
5 or more	0 (0.0)	1 (2.2)	
**Frequency of retching **^**a**^			
No	42 (93.3)	30 (66.7)	10.66, 0.01
1–2	2 (4.4)	6 (13.3)	
3–4	1 (2.2)	5 (11.1)	
5 or more	0 (0.0)	4 (8.9)	
**Amount of vomiting**			
Very large ^b^	0 (0.0)	0 (0.0)	12.53, 0.002
Large ^c^	0 (0.0)	0 (0.0)	
Moderate ^d^	0 (0.0)	5 (11.1)	
Small ^e^	0 (0.0)	6 (13.3)	
I did not throw up	45 (100.0)	34 (75.6)	
**Duration of nausea**			
Not at all	42 (64.4)	29 (64.4)	16.58, 0.001
1 h or less	3 (6.7)	2 (4.4)	
2–3 h	0 (0.0)	9 (20.0)	
More than 4 h	0 (0.0)	5 (11.1)	
**Administration of Anti-emetic drug in recovery room**			
Yes	0 (0.0)	4 (8.9) ^f^	4.18, 0.04
No	45 (100.0)	41 (91.1)	
**Administration of Anti-emetic drug in orthopedic ward**			
Yes	1 (2.4)	7 (15.9) ^f^	4.66, 0.03
No	41 (97.6)	37 (84.1)	

^a^times

^b^Very large (3 cups or more)

^c^large (2–3 cups)

^d^moderate (1/2–2 cups)

^e^small (up to 1/2 cup)

^f^Ondansetron was used

No significant difference was found between the groups regarding the neurovascular observation scale (vascular and neurological assessment and compartment syndrome signs) before the intervention. Moreover, the neurovascular observation scale was not different between the groups during the study period (*p* > 0.05) (Tables 3 and 4).

**Table 3:** Comparison of the neurovascular observation scale in the intervention and control groups during the study period

Neurovascular observation scale	Times	Groups								χ2, *p*—value
		Intervention				Control				
		Pink/normal	Pale	Dusky	Mottled/Purple	Pink/Normal	Pale	Dusky	Mottled/Purple	
Skin color	Before the intervention	36 (80.0)	1 (2.2)	4 (8.9)	4 (8.9)	27 (60.0)	4 (8.9)	9 (20.0)	5 (11.1)	5.12, 0.16
	Entering recovery room	37 (82.2)	1 (2.2)	4 (8.9)	3 (6.7)	30 (67.7)	1 (2.2)	5 (11.1)	9 (20.0)	3.84, 0.27
	4 h after entering the ward	38 (84.4)	0 (0.0)	4 (8.9)	3 (6.7)	31 (68.9)	0 (0.0)	5 (11.1)	9 (20.0)	3.84, 0.14
	8 h after entering the ward	39 (86.7)	0 (0.0)	3 (6.7)	3 (6.7)	31 (68.9)	0 (0.0)	5 (11.1)	9 (20.0)	4.41, 0.11
	12 h after entering the ward	41 (91.1)	0 (0.0)	3 (6.7)	1 (2.2)	39 (86.7)	0 (0.0)	3 (6.7)	3 (6.7)	1.05, 0.59
	16 h after entering the ward	45 (100)	0 (0.0)	0 (0.0)	0 (0.0)	41 (91.1)	0 (0.0)	2 (4.4)	2 (4.4)	4.18, 0.12
Skin Temperature		Normal	Hot	Heated	Cold	Normal	Hot	Heated	Cold	
	Before the intervention	38 (84.4)	0 (0.0)	0 (0.0)	7 (15.6)	32 (71.1)	0 (0.0)	0 (0.0)	13 (28.9)	2.31, 0.12
	Entering recovery room	28 (62.2)	0 (0.0)	0 (0.0)	17 (37.8)	24 (53.3)	0 (0.0)	0 (0.0)	21 (46.7)	0.72, 0.39
	4 h after entering the ward	42 (93.3)	0 (0.0)	0 (0.0)	3 (6.7)	41 (91.1)	0 (0.0)	0 (0.0)	4 (8.9)	0.15, 0.69
	8 h after entering the ward	45 (100)	0 (0.0)	0 (0.0)	0 (0.0)	45 (100)	0 (0.0)	0 (0.0)	0 (0.0)	It is not possible
	12 h after entering the ward	45 (100)	0 (0.0)	0 (0.0)	0 (0.0)	45 (100)	0 (0.0)	0 (0.0)	0 (0.0)	It is not possible
	16 h after entering the ward	45(100)	0 (0.0)	0 (0.0)	0 (0.0)	45 (100)	0 (0.0)	0 (0.0)	0 (0.0)	It is not possible
Pulse		Strong	Weak	Absent	Absent Doppler	Strong	Weak	Absent	Absent Doppler	
	Before the intervention	45 (100)	0 (0.0)	0 (0.0)	0 (0.0)	45 (100)	0 (0.0)	0 (0.0)	0 (0.0)	It is not possible
	Entering recovery room	45 (100)	0 (0.0)	0 (0.0)	0 (0.0)	44 (97.8)	1 (2.2)	0 (0.0)	0 (0.0)	1.01, 0.31
	4 h after entering the ward	45 (100)	0 (0.0)	0 (0.0)	0 (0.0)	45 (100)	0 (0.0)	0 (0.0)	0 (0.0)	It is not possible
	8 h after entering the ward	45 (100)	0 (0.0)	0 (0.0)	0 (0.0)	45 (100)	0 (0.0)	0 (0.0)	0 (0.0)	It is not possible
	12 h after entering the ward	45 (100)	0 (0.0)	0 (0.0)	0 (0.0)	45 (100)	0 (0.0)	0 (0.0)	0 (0.0)	It is not possible
	16 h after entering the ward	45 (100)	0 (0.0)	0 (0.0)	0 (0.0)	45 (100)	0 (0.0)	0 (0.0)	0 (0.0)	It is not possible
Movement of lower extremity fingers		Normal	Decreased	Limited	Absent	Normal	Decreased	Limited	Absent	
	Before the intervention	45 (100)	0 (0.0)	0 (0.0)	0 (0.0)	43 (95.6)	2 (4.4)	0 (0.0)	0 (0.0)	2.04, 0.15
	Entering recovery room	45 (100)	0 (0.0)	0 (0.0)	0 (0.0)	43 (95.6)	2 (4.4)	0 (0.0)	0 (0.0)	2.04, 0.15
	4 h after entering the ward	45 (100)	0 (0.0)	0 (0.0)	0 (0.0)	44 (97.8)	1 (2.2)	0 (0.0)	0 (0.0)	1.01, 0.31
	8 h after entering the ward	45 (100)	0 (0.0)	0 (0.0)	0 (0.0)	45 (100)	0 (0.0)	0 (0.0)	0 (0.0)	It is not possible
	12 h after entering the ward	45 (100)	0 (0.0)	0 (0.0)	0 (0.0)	45 (100)	0 (0.0)	0 (0.0)	0 (0.0)	It is not possible
	16 h after entering the ward	45 (100)	0 (0.0)	0 (0.0)	0 (0.0)	45 (100)	0 (0.0)	0 (0.0)	0 (0.0)	It is not possible
Sensation		Present	Decreased	Tingling/Numb	Absent	Present	Decreased	Tingling/Numb	Absent	
	Before the intervention	42 (93.3)	0 (0.0)	3 (6.7)	0 (0.0)	38 (84.4)	0 (0.0)	7 (15.6)	0 (0.0)	It is not possible
	Entering recovery room	45 (100)	0 (0.0)	0 (0.0)	0 (0.0)	45 (100)	0 (0.0)	0 (0.0)	0 (0.0)	It is not possible
	4 h after entering the ward	45 (100)	0 (0.0)	0 (0.0)	0 (0.0)	45 (100)	0 (0.0)	0 (0.0)	0 (0.0)	It is not possible
	8 h after entering the ward	45 (100)	0 (0.0)	0 (0.0)	0 (0.0)	45 (100)	0 (0.0)	0 (0.0)	0 (0.0)	It is not possible
	12 h after entering the ward	45 (100)	0 (0.0)	0 (0.0)	0 (0.0)	45 (100)	0 (0.0)	0 (0.0)	0 (0.0)	It is not possible
	16 h after entering the ward	45 (100)	0 (0.0)	0 (0.0)	0 (0.0)	45 (100)	0 (0.0)	0 (0.0)	0 (0.0)	It is not possible
Capillary filing		Under 2 s		Over 2 s		Under 2 s		Over 2 s		
	Before the intervention	44 (97.8)		1 (2.2)		44 (97.8)		1 (2.2)		It is not possible
	Entering recovery room	44 (97.8)		1 (2.2)		38 (84.4)		8 (15.6)		4.93, 0.02
	4 h after entering the ward	45 (100)		0 (0.0)		43 (95.6)		2 (4.4)		2.04, 0.15
	8 h after entering the ward	45 (100)		0 (0.0)		45 (100)		0 (0.0)		It is not possible
	12 h after entering the ward	45 (100)		0 (0.0)		45 (100)		0 (0.0)		It is not possible
	16 h after entering the ward	45 (100)		0 (0.0)		45 (100)		0 (0.0)		It is not possible

**Table 4:** Comparison of the signs of compartment syndrome in the intervention and control groups during the study period

The signs of compartment syndrome	Times	Groups								χ2, *p*—value
		Intervention				Control				
		**Yes**		**No**		**Yes**		**No**		
Pain on passive movement	Before the intervention	34 (75.6)		11 (24.4)		39 (86.7)		6 (13.3)		1.81, 0.17
	Entering recovery room	41 (91.1)		4 (8.9)		44 (97.8)		1 (2.2)		1.90, 0.16
	4 h after entering the ward	41 (91.1)		4 (8.9)		44 (97.8)		1 (2.2)		1.90, 0.16
	8 h after entering the ward	40 (88.9)		5 (11.1)		44 (97.8)		1 (2.2)		2.85, 0.09
	12 h after entering the ward	39 (86.7)		6 (13.3)		44 (97.8)		1 (2.2)		3.87, 0.04
	16 h after entering the ward	36 (80)		9 (20)		43 (95.6)		2 (4.4)		5.07, 0.02
Increasing pain not relived by analgesia		**Yes**		**No**		**Yes**		**No**		
	Before the intervention	2 (4.4)		43 (95.6)		1 (2.2)		44 (97.8)		0.34, 0.55
	Entering recovery room	6 (13.3)		39 (86.7)		7 (15.6)		38 (84.4)		0.09, 0.76
	4 h after entering the ward	9 (20.0)		36 (80.0)		11 (24.4)		34 (75.6)		0.25, 0.61
	8 h after entering the ward	3 (6.7)		42 (93.3)		5 (11.1)		40 (88.9)		0.54, 0.45
	12 h after entering the ward	1 (2.2)		44 (97.8)		11 (24.4)		34 (75.6)		9.617, 0.002
	16 h after entering the ward	0 (0.0)		45 (100)		1 (2.2)		44 (97.8)		1.01, 0.31
Pain at rest		**Nil**	**1–3**	**4–10**	**–––-**	**Nil**	**1–3**	**4–10**	**–––-**	
	Before the intervention	0 (0.0)	2 (1.1)	44 (97.8)	–––-	0 (0.0)	1 (2.2)	44 (97.8)	–––-	0.0, 1.0
	Entering recovery room	9 (20.0)	8 (17.8)	28 (62.2)	–––-	15 (33.3)	5 (11.1)	25 (55.6)	–––-	2.36, 0.30
	4 h after entering the ward	0 (0.0)	0 (0.0)	45 (100)	–––-	0 (0.0)	0 (0.0)	45 (100)	–––-	It is not possible
	8 h after entering the ward	0 (0.0)	0 (0.0)	45 (100)	–––-	0 (0.0)	0 (0.0)	45 (100)	–––-	It is not possible
	12 h after entering the ward	0 (0.0)	1 (2.2)	44 (97.8)	–––-	0 (0.0)	1 (2.2)	44 (97.8)	–––-	0.0, 1.0
	16 h after entering the ward	0 (0.0)	2 (4.4)	43 (95.6)	–––-	0 (0.0)	1 (2.2)	44 (97.8)	–––-	0.34, 0.55
Edema		**Nil**	**Mild**	**Moderate**	**Severe**	**Nil**	**Mild**	**Moderate**	**Severe**	
	Before the intervention	13 (28.9)	21 (46.7)	8 (17.8)	3 (6.7)	8 (17.8)	22 (48.9)	10 (22.2)	5 (11.1)	1.93, 0.58
	Entering recovery room	4 (8.9)	26 (57.8)	13 (28.9)	2 (4.4)	4 (8.9)	26 (57.8)	10 (22.2)	5 (11.1)	1.67, 0.64
	4 h after entering the ward	4 (8.9)	26 (57.8)	13 (28.9)	2 (4.4)	4 (8.9)	26 (57.8)	10 (22.2)	5 (11.1)	1.67, 0.64
	8 h after entering the ward	4 (8.9)	27 (60.0)	13 (28.9)	1 (2.2)	4 (8.9)	26 (57.8)	10 (22.2)	5 (11.1)	3.07, 0.38
	12 h after entering the ward	11 (24.4)	24 (53.3)	10 (22.2)	0 (0.0)	6 (13.3)	28 (62.2)	8 (17.8)	3 (6.7)	5.00, 0.17
	16 h after entering the ward	18 (40)	24 (53.3)	3 (6.7)	0 (0.0)	12 (26.7)	27 (60.0)	6 (13.3)	0 (0.0)	2.37, 0.30

## Discussion

This study showed that lemon inhalation aromatherapy reduced pain intensity in patients with lower extremity fractures upon entering the recovery room and at 4, 12, and 16 h post-surgery. In a mouse model study, the flavonoid eriocitrin in lemon fruit was found to have an antinociceptive effect on postoperative pain. This effect may be mediated by opioid and GABA receptors [14]. A systematic review and meta-analysis study indicated that aromatherapy reduced postoperative pain [21]. Another study which used lemon verbena extract showed this extraction reduced movement-induced pain and improved the muscle strength after exhaustive exercise [22]. In another study, it was reported that aromatherapy with orange as a genus of citrus reduced pain in patients with fractured limbs [23]. Citrus lemon may have an anti-nociceptive effect through central inhibitory mechanisms (opioid system) [15]. Moreover,, inhaled lemon essential oil reduced anxiety in healthy people [24] and patients with myocardial infarction [19]. This has anti-inflammatory properties, inhibits prostaglandin synthesis, and may play a role in redox-mediated mechanisms [15].

This study found that lemon inhalation aromatherapy reduced the severity and frequency of nausea, vomiting, and retching in patients who have undergone surgery for lower extremity fractures. This intervention also reduced the frequency of anti-emetic drug administration in postoperative lower extremity fracture patients. The clinical and important findings were that no patient in the lemon inhalation aromatherapy group needed anti-emetic drugs in the recovery room or 16 h postoperatively, respectively. Similarly, it was revealed that using aromatherapy with lavender, peppermint, ginger, and lemon reduced the number of orthopedic patients’ needs for anti-emetic drugs and their dose of anti-emetic administration [25]. In another study, the effect of lemon essence aromatherapy on reducing nausea and vomiting was shown in another study [26]. Moreover, in a study, it was mentioned that the combined lemon and peppermint aromatherapy decreased the intensity of nausea and vomiting [27]. Although the participants in above-mentioned studies were different from those in our study, the effect of lemon aromatherapy was reported in these studies.

As a cost-effective intervention, lemon inhalation aromatherapy might reduce pain, nausea, and vomiting in patients after lower extremity surgery. Therefore, this intervention had implications in practice.

The strength of this study was that the use of lemon essential oil was continued from the morning of the lower limb fracture surgery and extended at two-hour intervals until the end of the surgery, in the recovery room, and 16 h after surgery. Researchers’ assistant carefully carried out this intervention. Considering inclusion and exclusion criteria carefully minimized the effect of confounding factors, which was another strength of current study. To reduce the other confounders, ANCOVA was used and the type of anesthesia (general or spinal), use of analgesics in pre-,intra-, and post-operative stages, use or non-use of anti-emetic drugs in vomiting in recovery and in the post-operative ward were considered as confounding variables. Using random allocation and large randomized clinical trial was also another strength of this study.

Although the patients with lower extremity orthopedic surgery who underwent lemon essential oil indicated lower pain intensity, kind and dose of anesthesia medications might affect the pain feeling in the patients. As it was not possible to control all of these covariates, it was one of the limitations of this study. The second study limitation was related to inclusion criteria that patients with lower extremity fracture surgery (hip, femur, tibia, ankle, and heel) participated in this study. These various types and sites of fractures might affect the symptoms and treatment. Therefore, other studies are recommended to be conducted on one site of fracture, e.g., the hip, femur, tibia, ankle, and heel which received one type of treatment, e.g., open and close reduction and internal and external fixation of the foot.

## Conclusions

This study indicated that lemon inhalation aromatherapy reduced the pain intensity, post-operative frequency, and severity of nausea, vomiting, and retching. Moreover, this intervention reduced the amount of postoperative vomiting and duration of nausea. In addition, lemon inhalation aromatherapy decreased the frequency of anti-emetic drug administration in the recovery room and 16 h postoperatively. Therefore, using lemon inhalation aromatherapy, as a pain reliever and a reducer of nausea and vomiting, is suggested for lower extremity fracture cases who have undergone surgery. Conducting further studies to support evidence-based practice regarding pain, postoperative vomiting, and neurovascular and compartment syndrome of fractured patients who have undergone surgery is recommended.

## Data Availability

The datasets used and analyzed during this study is available from the first author on reasonable request using email to rambodma@yahoo.com.
